# *Mycobacterium marinum* Causes a Latent Infection that Can Be Reactivated by Gamma Irradiation in Adult Zebrafish

**DOI:** 10.1371/journal.ppat.1002944

**Published:** 2012-09-27

**Authors:** Mataleena Parikka, Milka M. Hammarén, Sanna-Kaisa E. Harjula, Nicholas J. A. Halfpenny, Kaisa E. Oksanen, Marika J. Lahtinen, Elina T. Pajula, Antti Iivanainen, Marko Pesu, Mika Rämet

**Affiliations:** 1 BioMediTech, University of Tampere, Tampere, Finland; 2 Department of Veterinary Biosciences, University of Helsinki, Helsinki, Finland; 3 Fimlab Laboratories, Pirkanmaa Hospital District, Tampere, Finland; 4 Department of Pediatrics, Tampere University Hospital, Tampere, Finland; McGill University, Canada

## Abstract

The mechanisms leading to latency and reactivation of human tuberculosis are still unclear, mainly due to the lack of standardized animal models for latent mycobacterial infection. In this longitudinal study of the progression of a mycobacterial disease in adult zebrafish, we show that an experimental intraperitoneal infection with a low dose (∼35 bacteria) of *Mycobacterium marinum*, results in the development of a latent disease in most individuals. The infection is characterized by limited mortality (25%), stable bacterial loads 4 weeks following infection and constant numbers of highly organized granulomas in few target organs. The majority of bacteria are dormant during a latent mycobacterial infection in zebrafish, and can be activated by resuscitation promoting factor *ex vivo*. In 5–10% of tuberculosis cases in humans, the disease is reactivated usually as a consequence of immune suppression. In our model, we are able to show that reactivation can be efficiently induced in infected zebrafish by γ-irradiation that transiently depletes granulo/monocyte and lymphocyte pools, as determined by flow cytometry. This immunosuppression causes reactivation of the dormant mycobacterial population and a rapid outgrowth of bacteria, leading to 88% mortality in four weeks. In this study, the adult zebrafish presents itself as a unique non-mammalian vertebrate model for studying the development of latency, regulation of mycobacterial dormancy, as well as reactivation of latent or subclinical tuberculosis. The possibilities for screening for host and pathogen factors affecting the disease progression, and identifying novel therapeutic agents and vaccine targets make this established model especially attractive.

## Author Summary

One third of the world's population has been estimated to be infected with *Mycobacterium tuberculosis*, which under the appropriate set of circumstances causes lethal lung disease. According to current understanding, mycobacteria can persist in their host without causing symptoms – a state referred to as latency or subclinical infection. However, if the immune system of the host becomes compromised, for example due to immunosuppressive medical treatments or HIV, the disease can become reactivated with detrimental consequences. The mechanisms leading to latency are not well understood. Latent tuberculosis responds poorly to antibiotics, and there is currently no effective vaccine against latent or reactivated tuberculosis. Using *Mycobacterium marinum*, a natural fish pathogen and a close relative of *M. tuberculosis*, we were able to induce a disease in adult zebrafish closely mimicking the human latent disease. We show that a dormant mycobacterial population is present in animals with a latent mycobacterial disease. Dormancy is also thought to occur in human tuberculosis. In addition, we present a method, with which the latent disease can be experimentally reactivated. Despite the evolutionary distance between man and fish, the zebrafish presents itself as a unique model for studying the mechanisms related to latency and reactivation.

## Introduction

Tuberculosis (TB) is caused by *Mycobacterium tuberculosis*, a highly specialized pathogen capable of evading the immune defense by various strategies. The success of the pathogen and the shortcomings of current medical interventions are reflected by the high prevalence of *M. tuberculosis* infection; one third of the world's population has been estimated to carry the pathogen and to have a latent, subclinical infection [1], which can be diagnosed using immunological sensitization to *M. tuberculosis* antigens [2]. Noteworthy, this asymptomatic infection is thought to consist of a variety of disease states that differ in bacterial phenotypes and burdens. [2], [3].

According to the report of the World Health Organization (WHO), TB caused 1.7 million deaths and 9.4 million new cases in 2009, especially in developing countries. Approximately 5–10% of carriers develop an active disease during their lifetime [4], which reflects the spectrum of disease states within the population with latent TB [2], [3]. This number is even higher in countries with a high prevalence of human immunodeficiency virus (HIV) [4]. The current preventive treatment against TB, the Bacille Calmette-Guérin (BCG) vaccine, protects children against the most severe forms of TB (TB meningitis or disseminated TB), but its efficacy in adults has been questioned and is thought to have limited or no protection against the disease [5], [6]. A worrisome shortcoming is that BCG does not protect against the reactivation of latent, subclinical TB [7]. The prevalence of HIV seems to be one of the most important attributes to the increase in the number of active TB cases [5], [8]. Tumor necrosis factor (TNF) neutralizing treatments often used in autoinflammatory diseases have also been found to increase susceptibility to TB [4], [5], as do malnutrition, tobacco smoke, indoor air pollution, alcoholism, insulin dependent diabetes, renal failure, and immune suppressive treatments, such as glucocorticoids [4]. These factors may either cause the primary infection to progress, or an existing subclinical infection to reactivate. In general, the mechanisms for the reactivation of tuberculosis are not well established and warrant further investigation.

Various animal models have been used for studying mycobacterial infections with the ultimate aim of understanding human TB [8]. The zebrafish has lately been established as a new, genetically tractable model for studying host–mycobacterium interactions [9]–[11]. Zebrafish are naturally susceptible to *Mycobacterium marinum* [12]–[14], which is a close relative of *M. tuberculosis* [15]. *M. marinum-*induced disease in zebrafish shares the main pathological and histological features, including necrotic granulomas, with human TB [16] and is thus a highly attractive model for the human disease. Zebrafish larvae have been widely used for studying innate immune responses to *M. marinum* infection [9], [11], [17]. However, adaptive immune responses have also been reported to be essential for controlling human TB [18], [19] and are also important for controlling *M. marinum* infection in adult zebrafish [10].

Studies on the latency, dormancy and reactivation of TB have been impeded by the lack of applicable animal models, as spontaneous latency without the help of chemotherapeutics has only been successful in the rabbit [20], and in macaque [21] models. Here, we show that a low-dose *M. marinum* infection spontaneously develops into a latent, non-progressive disease in adult zebrafish, with a static number of granulomas and a stable bacterial burden mainly consisting of dormant bacteria. The existence of a large dormant population of mycobacteria seems to be connected to the latent disease. In our model, the stable latent disease can be experimentally reactivated with γ-radiation, essentially mimicking the immune suppression-induced reactivation in human TB. This study thus presents a novel vertebrate platform suitable for large scale genetic screening, as a means of characterizing host and pathogen mechanisms underlying the transitions in TB from an acute infection to latency, and to a reactivated infection.

## Results

### A low-dose *M. marinum* infection leads to a latent disease with stable bacterial loads after 4 weeks

**Figure 1:**
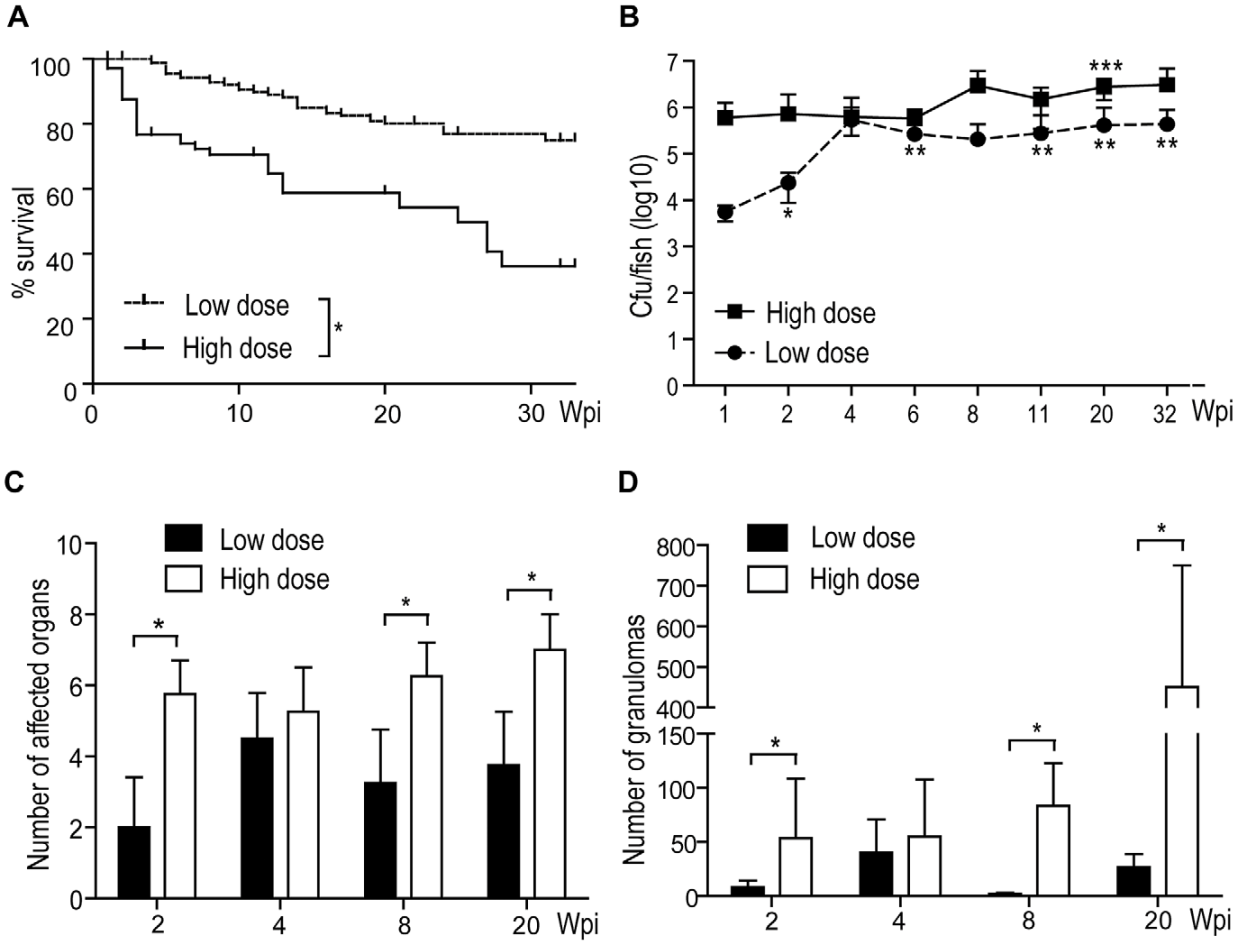
Zebrafish mortality, the development of bacterial load and the number of lesions have dose-dependent patterns. Adult zebrafish were i.p. infected with either a low (34±15 cfu) (n = 180) or a high dose (2029±709 cfu) (n = 104) of *M. marinum*. (A) Survival was followed for 32 weeks. * P<0.05 (B) The figure shows the average loads for 5 fish (except 32 wk high dose, n = 2). Low-dose statistics: * sig. diff. from 1 wk, ** sig. diff. from 1 and 2 wk. High-dose statistics: *** sig. diff. from 1, 2, 8, 11 and 20 wk. Low-dose vs. high-dose statistics: loads at time-points marked with † are sig. diff. (C) By default, 4 individuals per dose were analyzed by Ziehl-Neelsen staining (except 20 wk high dose, n = 3) per time-point The gonads, pancreas, liver, muscle, mesentery, spleen, gut and kidney were assessed and the number of organs with visible bacteria was determined. *P<0.05. (D) The total number of granulomas in a sample set for each individual was counted. * P<0.05.

The lack of suitable and well-established animal models mimicking latent, subclinical TB in humans prompted us to investigate if such a model could be developed in zebrafish. First, we compared several methods for infecting adult zebrafish with their natural pathogen, *M. marinum*, to create a physiological infection model leading to a static phase after the primary active disease. We infected zebrafish either by injecting different bacterial doses into the abdominal cavity or by bathing, to find a suitable dose and an infection route inducing a latent infection with low mortality. The experimental groups were followed up to 32 weeks for survival. A high-dose intraperitonaeal (i.p.) infection (2,029±709 cfu) was characterized by high mortality (end-point mortality 64%), whereas most fish infected with a low dose (34±15 cfu) generally survived (end-point mortality 25%) (Figure 1A). A group of fish was also infected with 9,075 ± 2,681 cfu, but this dose lead to an extremely high mortality (80% mortality in 5 weeks)(data not shown) and the group was excluded from further characterizations. Bathing the fish in water containing 2.4×10^6^ cfu/ml lead to an infection only in 50% of the individuals (determined by bacterial loads), which then developed a similar level of end-point mortality as the low-dose injected fish (data not shown). Because of the low incidence rate, bathing was not considered a suitable method for studying latent mycobacterial infection in adult zebrafish.

Latent human TB is diagnosed using tuberculin skin test (TST), interferon-γ release assays (IGRA) and characterized by a lack of clinical signs [2]. In our model, we are able to directly follow the progression of the disease by quantifying total mycobacterial burdens within the whole organism. For this purpose we developed a new, qPCR-based method specific for *M. marinum* (Supporting information, Text S1, Figure S1). In the high-dose group, an average bacterial load of 6.0×10^5^ cfu/fish (SD = 6.5×10^5^) was measured as early as 1 week post infection (wpi). Bacterial growth during the first week after injection was close to logarithmic, suggesting that the bacteria grew in an unrestricted manner. During the 32-week follow up, the average burdens rose to 3.0×10^6^ cfu/fish (SD = 3.2×10^6^), indicating that the high dose i.p. injection leads to a chronic progressive disease. Also in the low-dose group, the bacteria grew almost logarithmically during the first week of infection. The average bacterial load increased from the 1 weeks' 5×10^3^ (SD = 3.1×10^3^) to 4 weeks' 5.2×10^5^ cfu/fish (SD = 1.1×10^6^). After the four-week time point, however, the average bacterial burden ceased to grow, remaining at an unaltered level until the end of the experiment (at 32 weeks 4.4×10^5^ cfu ± 4.4×10^5^/fish) (Figure 1B). This result suggests that experimental infection of adult zebrafish by an i.p. injection of a small dose of *M. marinum* leads to an active primary infection, followed by a controlled state in most individuals.

### Granuloma formation and spreading of the infection ceases at the onset of the stable state infection in the low-dose infection model

**Figure 2:**
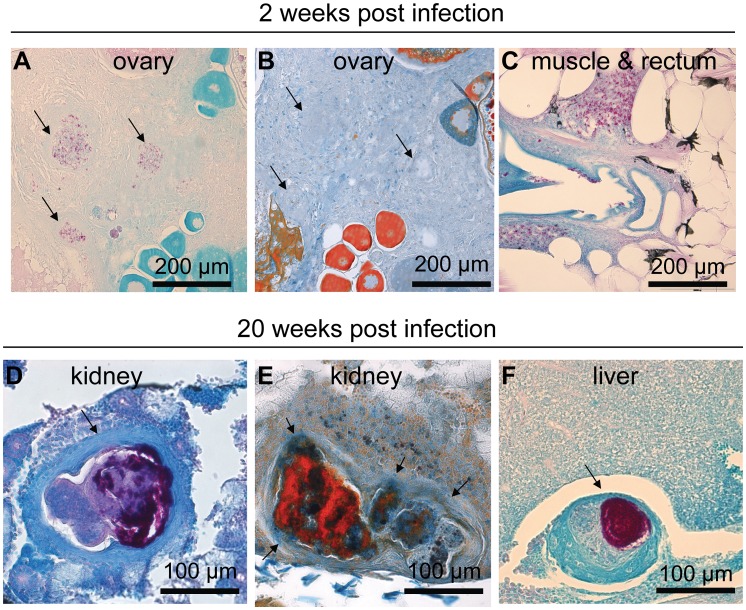
*M. marinum* induces the formation of granulomas that mature into well-defined structures during an infection. In fish infected with a low dose (34±15 cfu) of *M. marinum*, Ziehl-Neelsen staining at 2 wpi commonly reveals areas with free bacteria (C). Some slightly better formed and restricted areas containing bacteria, here referred to as early granulomas, are also seen (A), but as shown in (B) trichrome staining of the adjacent slide, encapsulation around the mycobacterial lesions is absent at the early stage of infection. At 20 weeks, fish that have survived have mature granulomas (D–F) many of which are multicentric surrounded by a fibrous capsule (D&E). (E) Trichrome staining shows the fibrous capsule in blue (F). The amount of bacteria inside granulomas has increased from the earliest time-points.

In order to get a more detailed and biologically relevant measure of the progression of the disease in our infection model, we carried out histological analyses at 2, 4, 8 and 20 wpi. Ziehl-Neelsen staining for mycobacteria was used for the quantification of granulomas and affected target organs. The gonads, pancreas, liver, muscle, mesentery, spleen, gut and kidney were specifically assessed for the presence of mycobacterial lesions. Early granulomatous structures characterized by cellular and bacterial aggregation were formed by 2 wpi in both dose groups (Figure 2A–D). The general appearance of the structures developed in the course of the infection such that at 20 weeks, most granulomas were insulated from the surrounding tissue by a fibrotic and/or cellular cuff (Figure 2E–H).

Granulomas were counted in representative sample sets for each individual (Figure 1D). Unsurprisingly, the fish infected with a low dose had significantly less granulomas at 2, 8 and 20 weeks following infection than the high-dose infected fish. The number of granulomas thus seems to be determined by the initial dose. In the high-dose infection, the number of granulomas significantly increased between 4 and 20 weeks, whereas in the low-dose infection, the number did not increase after the first 4 weeks, further supporting the relevance of our model for latent TB.

The number of affected organs was found to be determined by the initial infection dose. At 2 wpi, the low-dose infected fish had lesions in ∼2 organs (most often in the pancreas and gonads), whereas fish infected with the high-dose had bacteria in ∼6 organs (pancreas, kidney, gonads, liver, muscle, spleen). The number remained relatively unaltered for the duration of the experiment (Figure 1C), with the exception of a slight increasing trend in the high-dose group between 2 and 20 weeks. In the low-dose group, an increase between 2 and 4 weeks was seen (not significant), but the number of affected organs then ceased to grow, suggesting that the infection was well-controlled.

In conclusion, the histological analysis supports the idea that the high-dose infection is progressive with an increasing number of granulomas in various target organs, whereas the low-dose infection resembles a latent infection with unaltered numbers of granulomas in few target tissues.

### Cytokine responses to *M. marinum* differ between low-dose and high-dose infection

**Figure 3:**
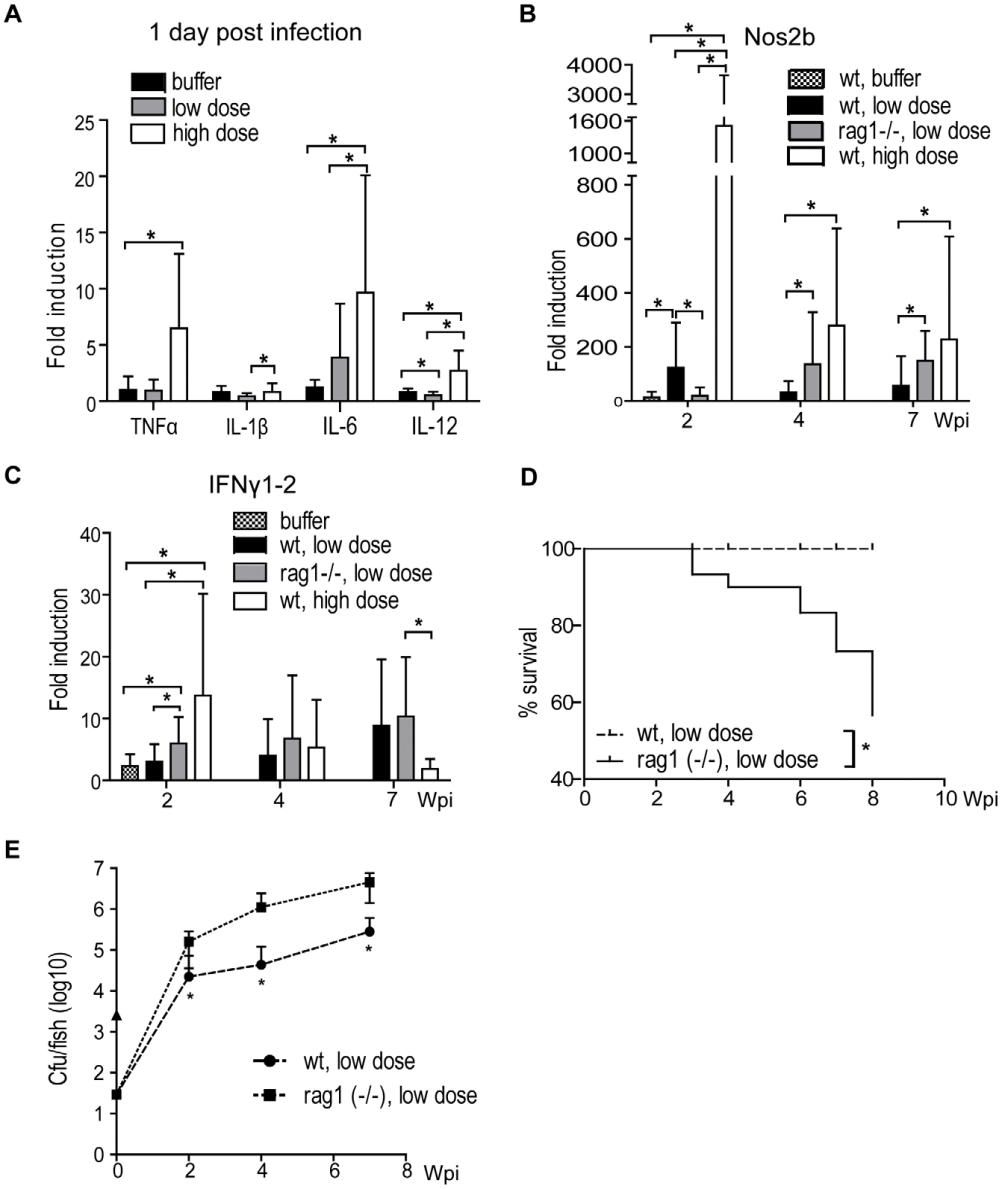
Bacterial dose and the presence of functional adaptive immunity define the outcome of mycobacterial infection. (A) The early cytokine response at 1 d post infection was measured from wt fish infected with a high (2029±709 cfu) or a low (34±15 cfu) dose or injected with sterile PBS buffer (n in each group 10–20). *P<0.05 (B) Wt fish were infected with a high or a low dose or sterile PBS buffer (for early time-points), and rag1 (−/−) fish were infected with a low dose Nos2b expression was measured with q-RT-PCR (n in each group was 9–20/time point). *P<0.05 (C) Fish were infected as in (B) and *IFNγ1–2* was measured with q-RT-PCR. *P<0.05. (D) Adult wt and rag1 (−/−) zebrafish were infected with a low dose (n = 30) and followed for survival. *P<0.05 (E) Adult wt and rag1 (−/−) fish were infected with a low dose. Average mycobacterial load was measured by qPCR at 2, 4, and 7 wpi (n = 10 per time point). *P<0.05.

To build a more detailed understanding on the different outcomes between the high and low dose infection, the early immune responses were studied by measuring cytokine expression levels in the internal organs of infected fish by reverse transcription quantitative PCR (q-RT-PCR). One day after infection, the high-dose infection caused an induction of *tumor necrosis factor alpha* (*TNFα, ZDB-GENE-050317-1*) by 6.5-fold (SD = 6.6), *interleukin 6* (*IL-6, ZDB-GENE-120509-1*) by 9.6-fold (SD = 10.4) and *interleukin 12* (*IL-12, ZDB-GENE-060724-1*) by 2.7-fold (SD = 1.8) (Figure 3C), but no induction was seen in *interleukin 1 beta* (*IL-1β, ZDB-GENE-040702-2*). Among the low-dose infected fish, only *IL-6* was induced but at a lower level, 3.9-fold induction, SD = 4.8, compared to high-dose infection at 1 dpi.

As the early innate responses are known to regulate the activation of adaptive responses, it was not surprising that differences in *interferon gamma 1–2* (*IFNγ1–2*, ZDB-GENE-040629-1) and *inducible nitric oxide synthase 2b* (*Nos2b*, ZDB-GENE-080916-1) levels were seen between the high and low dose groups at later time points (2–7 wpi). *Nos2b* was consistently more highly induced with the high dose than with the low dose at 2, 4 and 7 weeks (Figure 3D). The expression was at the highest level already at 2 wpi (high-dose group 1,508-fold, SD = 2,136, low-dose group 123-fold, SD = 167), after which the level declined in both dose groups, still remaining strongly induced.

In *IFNγ1–2* expression, the high dose caused a 13.7-fold induction (SD = 16) at 2 weeks. The low dose caused a more moderate 3.0-fold induction (SD = 2.8 (Figure 3F)), which was not different from the induction in the buffer-injected group. At 4 wpi, no difference was detected in *IFNγ1–2* levels. Noteworthy, at 7 wpi, the *IFNγ1–2* expression in the high-dose group had decreased to 1.8-fold induction (SD = 1.6), whereas in the low-dose group the level had increased to 8.8-fold (SD = 11.0), compared to uninfected controls. Thus, the kinetics of *IFNγ1–2* show a decreasing trend in the high-dose group and an increasing trend in the low-dose group, but the differences at late time-points are not significant. In conclusion, these results suggest that the strong early cytokine responses with the high infection dose are associated with *Nos2b* induction at an early phase of infection (2 wpi) and to the different kinetics of *IFNγ1–2* response between the two dose groups.

### Adaptive immunity is required for the restriction of bacterial growth and the induction of latency

According to the current understanding on human TB, adaptive immunity is required for efficient control of the disease [18], [19]. Survival results from a previous publication suggest a role for adaptive immunity in mycobacterial infection in the zebrafish [10]. We wanted to study whether adaptive immunity is required for the establishment of latency in the zebrafish. To this end, we used a recombination activating protein 1 (*rag1*) deficient zebrafish line, which lacks functional T and B cells [22].

First, we looked at the morbidity caused by a low dose of the type strain of *M. marinum* in rag1-mutant (−/−) zebrafish. Rag1 (−/−) fish, along with wild type (wt) controls, were infected with the low dose (34±15 cfu). The fish were euthanized at the end-stage of infection and survival curves were drawn (Figure 3D). None of the wt fish showed signs of disease during the 8-week follow up, whereas 43% of the rag1 (−/−) fish reached the end-stage of disease. DNA was extracted from the end-stage rag1 (−/−) fish and the mycobacterial load was measured by qPCR. The average load was 3.89×10^7^ cfu/fish (SD = 3.68×10^7^), which is similar to the levels measured from terminal stage *M. marinum* infected wt fish (data not shown), indicating that the rag1 (−/−) zebrafish had suffered from an end-stage *M. marinum* infection.

Dynamic disease progression among rag1 (−/−) fish was associated with elevated mycobacterial loads compared to wt controls during the first weeks of infection. Rag1 (−/−) and wt fish were infected with the low dose for determination of bacterial burdens by qPCR. Already at 2 wpi, the loads in the rag1 (−/−) fish were significantly higher (1.61×10^5^ cfu/fish, SD = 1.25×10^5^) than in the wt fish (2.22×10^4^ cfu/fish SD = 4.99×10^4^), indicating that the adaptive immune responses are used already by 2 wpi as a means of restricting the mycobacterial infection. During the following weeks, the bacterial burdens remained significantly higher in the rag1 (−/−) mutants (3.80×10^6^ cfu, SD = 3.15×10^6^) compared to wt fish (2.83×10^5^ cfu, SD = 3.26×10^6^ at 7 wpi).

Alongside with gene-expression measurements from wt fish, *Nos2b* (Figure 3B) and *IFNγ1–2* (Figure 3C) levels were measured from low-dose infected rag1 (−/−) fish. At 2 wpi, *Nos2b* expression was significantly lower in rag1 (−/−) fish (19.6-fold, SD = 30.3) compared to the wt fish (123-fold, SD = 16), suggesting that adaptive responses affect *Nos2b* induction during the early phase of infection preceding the latency. It is generally thought that in human TB, *Nos2* is induced as a result of IFNγ production by lymphocytes, leading to macrophage activation and control of mycobacterial growth. However, in the adult zebrafish model the *Nos2b* induction at 2 wpi is not likely to be mediated by an adaptive *IFNγ1–2* induction, as the measured *IFNγ1–2* levels were significantly higher in the rag1 (−/−) mutants (6.0-fold induction, SD = 4.5) than in the wt fish (3.0-fold induction, SD = 2.8). At 4 and 7 weeks, the situation was altered so that the rag1 (−/−) mutants had significantly higher *Nos2b* expression levels (induced 136-fold, SD = 193 and 149-fold, SD = 110, respectively) than those observed in the wt (induced 31.6-fold, SD = 42.0 and 56.6-fold induction, SD = 108, respectively). These results suggest that in the adult zebrafish model, the initial macrophage activation preceding the onset of latency is mediated by adaptive responses driving Nos2b induction, but unexpectedly, not via IFNγ.

### Most mycobacteria enter a dormant state during a latent infection in adult zebrafish

In human TB, the majority of bacteria are thought to enter a dormant state in response to the stress caused by the immune response and hypoxia. Dormant bacteria are viable but not culturable (VBNC) [23]. This state has been shown to be reversible by the addition of a resuscitation promoting factor (Rpf) *in vitro* [24]. The role of dormancy and resuscitation in a latent mycobacterial infection is difficult to study in humans, as the putative dormant bacteria are not accessible for visualization and cannot be cultured [23]. To investigate, whether there is a dormant bacterial population in *M. marinum* infected adult zebrafish, we tested the effect of Rpf on the number of colonies cultured from fish with a latent infection.

**Figure 4:**
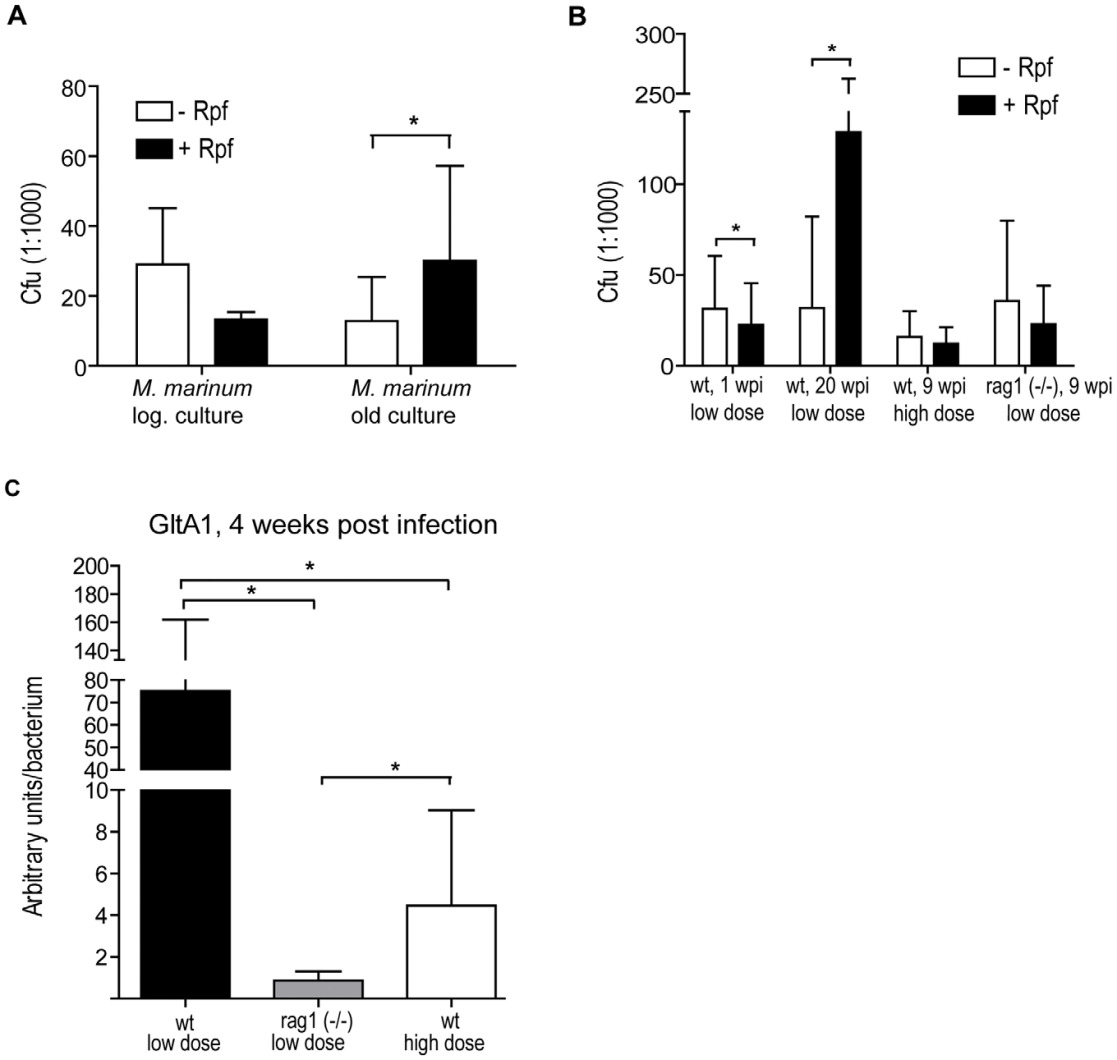
A major part of the mycobacteria are in a dormant state in latent infection. (A) Parallel dilutions of fresh logarithmic or old plateau phase *M. marinum* cultures were plated +/− Rpf to show the resuscitating effect of *Micrococcus luteus* Rpf on dormant *M. marinum*. (B) Parallel homogenate sample dilutions from low-dose (34±15 cfu) infected fish (wt or rag1 (−/−)) were plated at different time points +/− Rpf to detect dormant mycobacteria. (C) *GltA1* expression was measured from low-dose infected rag1 (−/−) and wt fish and high-dose infected wt fish and normalized to the total *M. marinum* load in each fish measured by qPCR. *P<0.05.

First, we tested if hypoxic *M. marinum* cultures can be resuscitated by an addition of *Micrococcus luteus* Rpf on antibiotic plates. Of note, the standard method of assessing the effect of Rpf on mycobacterial growth in broth culture and most probable number assay could not be used due to the fast-growing contaminating normal flora from the gut. Dilutions of active logarithmic and old hypoxic *M. marinum* broth cultures were plated with and without Rpf. As expected, Rpf significantly increased the number of colonies plated from old, hypoxic, inactive cultures (2.4-fold increase) but did not increase the number of colonies of active bacteria (Figure 4A). Altogether, these results indicate that Rpf from *M. luteus* media is active on 7H10 plates and is able to cause resuscitation of a significant proportion of dormant *M. marinum* that do not otherwise grow on culture plates. This also confirms the role of Rpf as a resuscitating enzyme for *M. marinum*, resembling its well established function for *M. tuberculosis*.

Next, adult zebrafish were infected with the low dose, and the disease was allowed to develop for twenty weeks before the fish were collected for analysis. Parallel samples were analyzed in the presence and absence of Rpf on the plate. When the diluted samples from fish with a latent infection were plated in the presence of Rpf, the number of culturable *M. marinum* increased 4-fold (32 ± 50 cfu without Rpf compared to 129 ± 134 cfu with Rpf) (Figure 4A). For early infection stage samples (1 wpi), the addition of Rpf did not have a growth promoting effect (31 ± 29 cfu without Rpf, 21 ± 22 cfu with Rpf) (Figure 4). With the high infection dose, leading to a more progressive disease, the population of resuscitable dormant bacteria were not detected at 9 wpi using Rpf (Figure 4A). Similarly, in the low-dose infected rag1 (−/−) fish, Rpf did not increase the average number of culturable mycobacteria, suggesting that adaptive immunity has a role in the efficient induction of mycobacterial dormancy. These results indicate that a distinguishable dormant mycobacterial population exists in the zebrafish with a latent infection, whereas in the active infection bacteria are predominantly in a replicative form.

To further confirm the existence of dormant mycobacterial population in the zebrafish with a latent infection, we measured the expression levels of known dormancy-associated mycobacterial genes. Based on *M. tuberculosis in vitro* dormancy microarray data [25], *HspX* (MMAR_3484), *devr* (MMAR_1516), *tgs1* (MMAR_1519) and *GltA1* (MMAR_1381) were chosen for q-RT-PCR measurements. Of these, only *GltA1*, which encodes a metabolic enzyme called citrate synthase, had generally high enough expression levels for reliable quantification from fish with a latent infection. *GltA1* expression was measured at 4 wpi from high-dose infected wt fish and low-dose infected wt and rag1 (−/−) fish. The *GltA1* expression level normalized to the number of bacteria in the low-dose infected wt fish (75.2, SD = 86.8) was significantly higher than in the high-dose wt fish (4.46, SD = 3.55), supporting the idea that in latent infection the proportion of dormant mycobacteria is greater than in a more progressive infection. The lowest *GltA1* expression/bacterium was seen in the low-dose infected rag1 (−/−) fish (0.86, SD = 0.44). The low *GltA1* expression in rag1 (−/−) fish, together with the plating result showing no resuscitating effect by Rpf in rag1−/− fish (Figure 4B), suggests that adaptive immunity plays a role in the induction of mycobacterial dormancy *in vivo*.

### The reactivation of a latent mycobacterial infection in zebrafish can be induced by γ-irradiation

**Figure 5:**
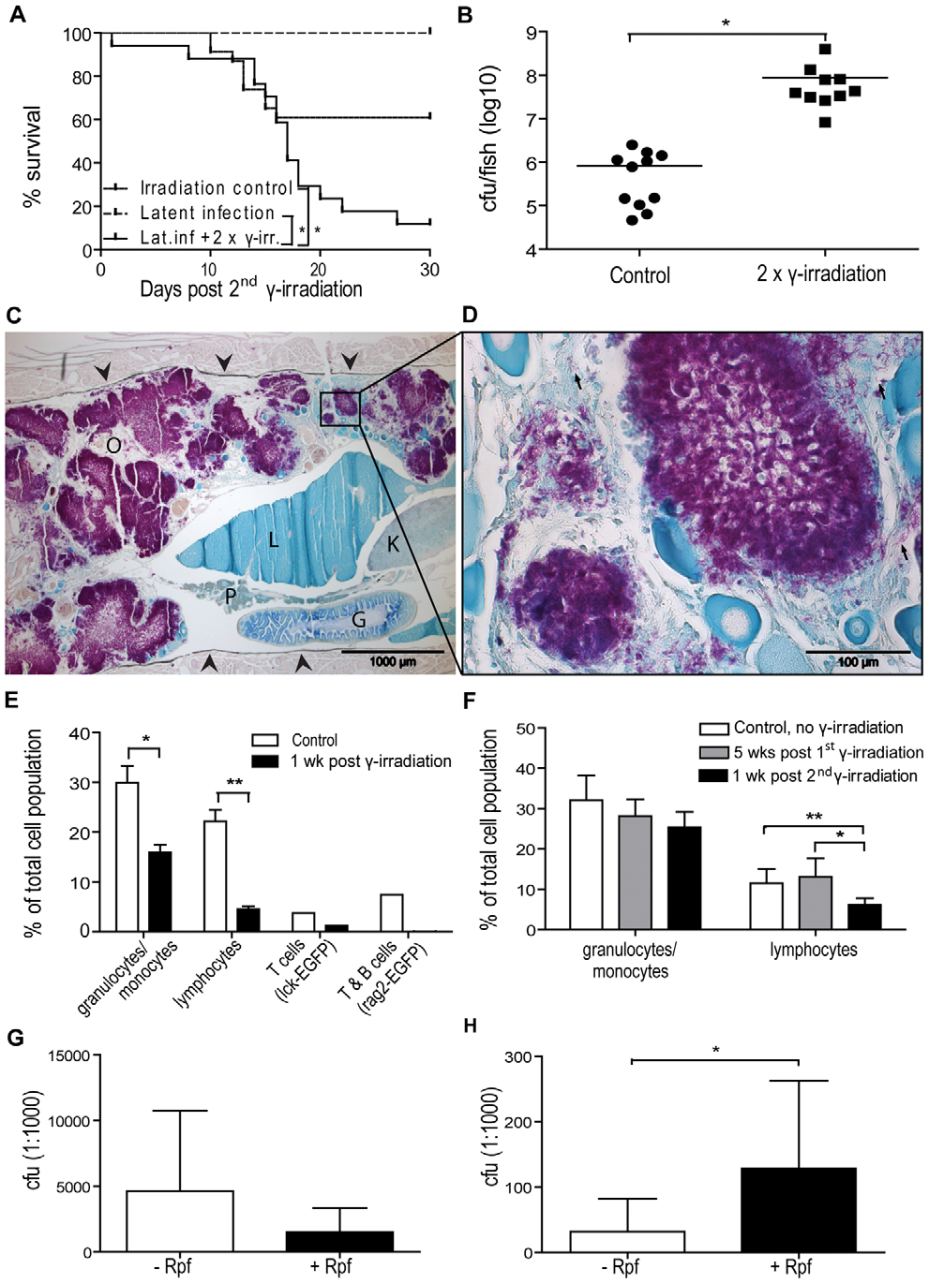
Gamma irradiation induces reactivation resulting in increased mortality due to uncontrolled growth of mycobacteria. (A–C) Zebrafish (n = 17) with a latent *M. marinum* infection were irradiated twice with 25 Gy with one month between the irradiations. Twice irradiated, non-infected zebrafish (n = 23) as well as zebrafish with a latent infection (n = 14) were included as controls. (A) Survival was followed for 30 days after the second dose. *P<0.05. (B) During this period, moribund or recently dead fish were collected 15–22 days after the second radiation dose. Bacterial loads were compared with those of similarly infected, non-irradiated control fish that were collected at the end-point of the experiment. *P<0.05 (C&D) A representative Ziehl-Neelsen stained sample from a reactivated fish showing large numbers of free mycobacteria (purple areas) in the zebrafish body cavity (C). The sides of the body cavity are marked with arrowheads O = ovary, P = pancreas, L = liver, G = gut, K = kidney. (D) A picture taken with a higher magnification showing individual rods (few examples pointed out with arrows). (E) Four groups of 4 adult zebrafish (1 rag2-gfp, 1 lck-gfp and 2 wild-type groups) were γ-irradiated with 25 Gy. Similar control groups were left untreated. Kidneys were collected 8 d post irradiation, pooled and analyzed by FCM. FSC-SSC -plots were gated based on cell size and granularity as described in [56] (gates shown in Figure S3) to assess the effect of irradiation on leukocyte populations. *P<0.05. For further verification of the effect of radiation on lymphocytes, a GFP gate was used for the rag2 and lck groups expressing GFP in B and T cells, or T cells, respectively. (F) Adult non-infected wt zebrafish were irradiated with 25 Gy once (grey bars) (n = 3) or twice (n = 7) (black bars) with one month between the doses. Leukocyte recovery and re-depletion were assessed by FCM. Non-irradiated fish (n = 4) were used as controls. *P<0.05 (G) Fish with a latent infection (n = 7) were irradiated twice with 25 Gy with one month between the doses and plated +/− Rpf for 18 d after the second radiation dose. (H) Fish (n = 6) with a latent infection were plated +/− Rpf.

Various immunosuppressive medical treatments, such as glucocorticoids [4] and radiation treatment [26], are seen as factors that increase the risk of the reactivation of latent human TB. Having established a model for latent mycobacterial infection in adult zebrafish, we next moved on to test the effect of γ-irradiation as immunosuppressive treatment to reactivate latent mycobacterial infection. Fish were infected with the low dose (34 ± 15 cfu), and five months post infection, a group of fish was irradiated with 25 Gy. Survival was followed for 1 month post irradiation, and the bacterial load was determined at 2 weeks. As a single 25 Gy dose of γ-radiation did not seem to cause sufficient reactivation of the latent mycobacterial infection in our zebrafish model system (Figure S2), two 25 Gy doses were administered to a group of fish with a latent *M. marinum* infection with one month between the doses. Survival was followed for one month after the second irradiation. To assess the changes in the mycobacterial numbers and lesions, moribund or recently dead fish were collected and analyzed either histologically or with *M. marinum-*quantification PCR. Two 25 Gy doses of γ-radiation caused some degree of early time-point mortality in both irradiated groups. However, in the non-infected group, no deaths occurred after 16 days from the second irradiation (total mortality 40%), whereas the infected, irradiated population continued to die, reaching an end-point mortality of 88% (Figure 5A). No deaths occurred in the non-irradiated latent infection group. The immunosuppressive treatment with two 25 Gy doses of γ-irradiation lead to a significant increase in mortality among zebrafish with a latent mycobacterial infection, suggesting reactivation of the disease.

To confirm that the increased mortality after the γ-irradiation was related to the progression of the mycobacterial infection, the bacterial burdens were determined. Fish collected for qPCR 15–22 days after the second γ-radiation dose had an average bacterial load of 8.7×10^7^ cfu (SD = 1.2×10^8^), which was 106-fold higher compared to non-irradiated controls (average load 8.2×10^5^ cfu, SD = 8.1×10^5^) (Figure 5B). A histological analysis of moribund individuals revealed vast areas of free bacteria not restricted to granulomas (Figure 5C,D). Based on these results, γ-irradiation-induced reactivation of latent mycobacterial infection in adult zebrafish is a highly promising model for investigating the cellular and molecular mechanisms involved in reactivated mycobacterial infections.

### Gamma irradiation-induced depletion of lymphocyte populations is associated with the reactivation of latent mycobacterial infection

To characterize the effect of γ-irradiation on blood cells, the changes in different blood cell populations were analyzed using flow cytometry (FCM). The numbers of granulo/monocytes and lymphocytes were measured from kidney homogenates. First, the immediate effects of a 25 Gy dose of γ-irradiation were studied by analyzing changes one week after the treatment (Figure 5E). The average proportion of granulocytes and monocytes was reduced by 47%, however there was a striking 80% reduction in the lymphocyte population, compared to normal levels. The efficient depletion of lymphocytes was further verified using the fish lines Tg(lck:lck-EGFP) and Tg(rag2-GFP), which express GFP in T cells, or in T and B cells, respectively. With these fish, a 67% reduction in the T cell population (lck) and a 99% reduction in the B and T cell population (rag2) were seen one week after irradiation (Figure 5E). Despite the marked leukocyte depletion, one 25 Gy dose of γ-irradiation had not been sufficient for the reactivation of a latent mycobacterial infection in zebrafish, as no significant changes were seen in mortality rates (Figure S2A) or in bacterial burdens (Figure S2B). Therefore, we next studied the recovery of leukocytes after the first irradiation, as well as the short- term effect of the second 25 Gy dose (Figure 5F). Both lymphocyte and granulocyte/monocyte populations had recovered to normal levels by five weeks after the first 25 Gy dose. The second 25 Gy dose of γ-irradiation reduced the number of lymphocytes by 53% compared to the recovery levels (Figure 5F), whereas granulocytes were not significantly affected by the second treatment. These results suggest that the effective reactivation of a latent mycobacterial infection required two 25 Gy doses of γ-irradiation because of the rapid recovery of the lymphocyte and granulocyte/monocyte populations after the first treatment. In addition, the mechanism of reactivation in this model is most likely due to the specific depletion of lymphocytes rather than a decrease in granulocytes.

### Immunosuppression by γ-irradiation leads to reactivation of the dormant mycobacterial population

To assess the changes in the dormant bacterial population after the reactivation, we plated samples in the presence and absence of Rpf at 2.5 weeks after the second 25 Gy irradiation dose. In the non-irradiated fish with a latent infection, the number of colonies were 4-fold higher in the presence of Rpf than in its absence (Figure 5H), whereas after double irradiation the resuscitating effect of Rpf could no longer be seen (Figure 5G). This result supports the idea of latency-associated mycobacterial dormancy, which is reversed in reactivated disease.

## Discussion

During the last couple of decades, the prevalence of active TB has substantially increased. Many of these cases are likely to be due to the reactivation of latent TB as a consequence of various immune compromising factors, such as HIV [27], diabetes [28] and glucocorticoid treatment [29]. Currently, the reactivation of latent TB is one of the greatest challenges in the field of infectious diseases, as present vaccination strategies do not protect against this phase of infection [7]. The fact that multiresistant strains of *M. tuberculosis* are arising in many parts of the world [5], [30] further complicates the control of this disease. Thus, more detailed information on the mechanisms of the host–pathogen interactions in a latent mycobacterial disease and its reactivation is indispensable.

In general, the *M. marinum* infection model in zebrafish is well established. As *M. marinum* is a common pathogen of zebrafish, it can be considered a more natural model for studying host–mycobacterium interaction, than is, for example the *M. tuberculosis* mouse model. The histopathology of mycobacterial lesions in zebrafish has been shown to be more similar to human TB than is the histopathology in the mouse model (reviewed in [14]). The genetic similarities between *M. marinum* and *M. tuberculosis* are well documented [15], including the currently known genes involved in virulence and in dormancy (Dos-regulon) [31]. Thus, it is likely that the characterization of phenomena involved in latent infections and dormancy in a *M. marinum* infection, is useful for understanding human latent TB.

The concept of latent TB is problematic, and a debate over the definition as well as the nature of latent TB is on-going [32]. “Latent TB” is a broad clinical definition diagnosed with indirect immunological reactions in the tuberculin skin test (TST) or the interferon-γ release assay (IGRA) in the absence of clinical symptoms [2]. These assays do not reveal whether there are viable bacilli present in the host, but rather, whether the host has been infected with the bacterium and developed an adaptive response against it. Thus, cases diagnosed with latent TB compose a heterogeneous group with different bacterial phenotypes and loads [2], [3]. In studies on latent TB patients, DNA of *M. tuberculosis* has been shown to be generally present in the lung necropsy samples of individuals with a latent infection [33], [34]. These findings are in harmony with the common latency paradigm stating that in most infected individuals mycobacteria become dormant and non-replicating in the hypoxic environment of the granuloma but can be resuscitated in non-restrictive circumstances [2]. Still, the presence of mycobacterial DNA, as such, does not reveal the metabolic status (dormancy) of the bacteria. The subject warrants further investigation in applicable animal models as well as in human cohorts.

In this study we set up a novel model for latent TB using experimental *M. marinum* infection of adult zebrafish. We showed that mycobacterial dormancy is a central feature of latent TB in the zebrafish. The importance of adaptive immunity in the establishment of a latent disease in zebrafish was shown in a number of experiments carried out with rag1 (−/−) zebrafish that lack T and B cells. In addition, we developed a pioneering adult zebrafish model, in which an immunosuppressive radiation treatment was used for reactivation of the latent disease. With this model, various aspects of the currently poorly characterized process of latency, dormancy and reactivation can be studied in a simple vertebrate system.

As a first step, we had to be able to induce a non-progressive, but persistent, infection in adult zebrafish. Based on previous work in adult zebrafish, the severity of the disease is dependent on both the dose and the strain [10], [35], [36]. The type strain of *M. marinum* (ATCC 927) has previously been reported to produce a moderate infection in zebrafish, but previously only high doses have been used [35]. In our hands, a low dose of this strain delivered as an injection (i.p.) was found to be the most reliable means of inducing a latent infection. In addition to injecting, bathing in water infested with different concentrations of mycobacteria was also tested. Although bathing could provide a more natural route of infection through the gills or the gut, the low incidence rate achieved by this method made it unsuitable for this study. As our scope is to study latency and reactivation and not the natural course of initial colonization, i.p. injection was considered applicable for our purposes.

The non-progressive status of the experimental infection could be verified by quantifying bacterial loads in the fish using an in-house-developed qPCR assay, and by quantifying granulomas in full-length longitudinal sections. Most fish did not show any signs of disease, and the average bacterial burdens as well as the number of granulomas and affected organs cease to grow after 4 weeks of infection remaining at a static level in the majority of individuals. This essentially demonstrates the central features of the latent disease. The disease is present in the host and has the potential to reactivate under appropriate circumstances. A centrally important feature of our model is that the non-progressive state developed naturally between the host and the mycobacteria without further intervention, and lasted for the entire duration of the 8-month study in 75% of the individuals.

The results gained with the quantitative PCR method in our model showed that the total number of mycobacteria ceased to increase after the first weeks of infection and remained stable for the entire duration of the study. Bacteria entering a non-replicating, dormant state would be a reasonable explanation for the non-progressive bacterial burdens; which is also thought to happen in human TB. To examine whether the bacteria entered a dormant state in our model system, we carried out *ex vivo* plating experiments. Comparing the efficacy of *ex vivo* growth in liquid broth and solid plate has been previously used for showing dormant *M. tuberculosis* populations in chronically infected mice [37]. We used an alternative, specific method using resuscitation promoting factor (Rpf) from *Micrococcus luteus*. Rpf has been shown to resuscitate dormant *M. luteus* but also various mycobacterial species [24]. Homologous proteins with the same function have thereafter also been found to be present in actively dividing mycobacterial cultures [38], and the functions of these muralytic enzymes has been extensively studied in mycobacterial species [39]. Mutant *M. tuberculosis* strains without functional Rpfs have been shown to be less virulent and unable to reactivate *in vivo* [40], [41].

Using *M. luteus* Rpf on solid plates, we found that the majority of the bacteria in most fish with a latent infection were actually in a dormant, viable but not culturable state, and could be resuscitated by the addition of Rpf. The resuscitable population of dormant mycobacteria seen in latent wt fish was absent in rag1 (−/−) fish lacking functional adaptive immunity. Also, the expression level of the known dormancy-associated enzyme, citrate synthase (*GltA1*), in wt fish was 87-fold higher than in the rag1 (−/−) fish, indicating that effective induction of mycobacterial dormancy is mediated by adaptive immune responses. The presence of Rpf on plates did not increase the number of culturable mycobacteria in samples representing the active phases of infection; namely the primary active disease with a low dose, a progressive disease with a high dose and the reactivated infection. These results suggest that dormancy of a high proportion of the total mycobacterial population is associated with the latent disease. In this study, there was variation in all the measured parameters within the experimental grou25ps with latent infection. This variation is most likely explained by differences in disease progression between individuals within the latent groups. Similar wide disease spectrum is thought to be present also in the human latent TB [2], [3]. To characterize the underlying factors leading to this typical variation in disease outcomes, it would be beneficial to follow the disease progression in individuals instead of heterogeneous groups in studies using *in vivo* models of TB.

The early cytokine responses (*TNFα, IL-6, IL-1β, IL-12*) were measured on the first day of low-dose or high-dose infection. The high dose generally evoked a stronger pro-inflammatory response, which may have contributed to the high mortality in the beginning of the infection. Conversely, the low-dose infection seemed to avoid evoking strong responses. Of the measured cytokines, only *IL-6* was induced. IL-6 has been reported to be important in restricting mycobacterial growth [42] and in efficient protection by vaccination against TB in mice [43], and as such, may have had a role in the initiation of a latent disease in the zebrafish.

The differences in the disease progression were further studied at later time-points, where *Nos2b* and *IFNγ1-2* expression levels were measured. According to current hypothesis, IFNγ induces Nos2 in macrophages activating them to more efficiently destroy intracellular mycobacteria [44]–[46]. In our zebrafish model, *Nos2(b)* was clearly induced with both the low and the high dose at 2–7 wpi. In the high-dose infection group, the induction at 2 weeks was as high as ∼1500-fold compared to baseline levels. Despite this strong induction, most of the fish succumbed to infection, perhaps due to insufficient phagocytic capacity. At the same time, *Nos2b* was not induced in rag1−/− fish at 2 wpi, and the bacterial burdens were already significantly higher than in the wt low dose animals. Based on this, adaptive responses mediate the *Nos2b* induction and are required for the restriction of mycobacterial growth already at this stage. However, the adaptive mechanism behind this induction in the mycobacterial disease in the zebrafish remains obscure, as *IFNγ1-2* was not induced at 2 weeks in the low-dose infected wt fish. Later on, at 4 and 7 wpi, an induction of Nos2b was also seen in rag1 (−/−) fish, indicating that the innate arm of immunity alone, to some extent, can induce the production of nitric oxide as a response to the high bacterial numbers.

According to the latest hypotheses on latent TB, the grand scheme is complex with various co-existing populations of mycobacteria in different niches and metabolic states. Some of these populations have been suggested to constantly probe the environment in search of prospects for reactivation (e.g. immunodeficiency), whereas others are in a less active state, waiting for resuscitation signals from the probing population. The proportion of bacteria in each population determines the disease status. Likely, a fully functional immune system is able to keep this small active population in line. In case of immunosuppression, the active population replicates and excretes resuscitation factors, leading to reactivation of the dormant population [23]. Our findings in the zebrafish model support this elegant hypothesis.

Latent mycobacterial infection models have previously been set up in the rabbit [20], in the mouse [47], [48], in the guinea pig [49] and in the macaque [21]. Some evidence on bacterial dormancy exists in the chronic disease of vaccinated mice [37]. The rabbit and macaque models were induced with a low bacterial inoculate similarly to our zebrafish model, whereas the mouse and guinea pig models utilize chemotherapeutics. The historical Cornell's model of latency in the mouse is artificially induced by antibiotic treatment. After the antibiotics are removed, a spontaneous reactivation occurs [47]. Without antibiotic treatment, the bacterial loads continue to increase, eventually leading to the death of the mouse. Others have utilized streptomycin auxotrophic strains of *M. tuberculosis* whose growth can be arrested in the absence of streptomycin, resulting in a paucibacillary state [48], [49]. However, in our zebrafish model, the infection is induced with a naturally replicating strain and generally results in a latent disease without any antibiotic treatment. Thus, it is likely that the mycobacterial infection in the zebrafish more accurately models the natural course of infection that is determined by the interplay between the pathogen and the immune response of the host. In our model, as many as 75% of the fish survived the 8-month period and managed to restrict further bacterial growth, suggesting that a latent-type disease developed. The percentage of latent-type cases was much higher in zebrafish than in the macaque model (40%) [21]. In humans, 90–95% of TB cases are subclinical [4].

Having established a potential model for latent TB, we next set up a method for reactivation *in vivo*. So far, reactivation has only been established in non-human primate models in the context of simian immunodeficiency virus (SIV) [50], in a rabbit model with dexamethasone [20] and in a murine model with aminoguanidine [51]. Even though these models are likely to replicate the human TB-HIV co-infection and glucocorticoid-induced reactivation, respectively, the zebrafish could provide a useful and ethical model for large-scale experiments on reactivation. To our knowledge, irradiation has not been previously used for inducing reactivation of latent mycobacterial infections.

First, we tested whether irradiation could be used for the reactivation of the latent disease. Surprisingly, despite irradiation killing almost all lymphocytes and half of the granulo/monocytes, a single dose (25 Gy) was found to be insufficient for a general reactivation of the mycobacterial disease during the one-month follow-up period. This could be due to the combined effect of the rapid recovery of the leukocyte population after irradiation [52] and the low growth rate of mycobacteria. In adult zebrafish, leukocyte numbers have been reported to recover to pre-irradiation levels in 2 weeks after a 20 Gy dose [52]. However, when the 25 Gy was administered to latently infected fish twice, with one month between the doses, the desired effect on the mycobacterial disease was achieved. Mortality increased significantly compared to latently infected controls and similarly irradiated healthy fish. A descriptive histological analysis of moribund individuals also revealed vast areas of free bacteria outside granulomas. The mycobacterial loads in twice irradiated fish with end-stage infection had increased by ∼100-fold compared to stable state levels. The kinetics of the bacterial outgrowth in these fish is in harmony with theoretical calculations of unrestricted bacterial growth. Leukocyte numbers have been reported to reach the lowest level 6–7 days after 20 Gy of radiation [52]. At this point, the bacteria should be able to grow without limitation. In liquid culture at 29°C, the *M. marinum* used in our laboratory doubles its numbers in 24 hours and thus a 100-fold increase would require ∼7 days. Indeed, in the reactivation group, a steep drop in survival after 16 days concomitant with high bacterial loads was seen. Based on these results, the zebrafish model for the reactivation of a mycobacterial disease appears highly promising.

Radiation treatments, as is well known, have various biological effects. When considering irradiation as a method for reactivating a mycobacterial infection, some of these effects need to be discussed. Firstly, the dose used in this study (25 Gy) is high and would be lethal for mammals. Zebrafish seem to be relatively resistant to the acute adverse effects caused by irradiation, as the treatment *per se* did not cause mortality. The lethal dose for adult zebrafish has been reported to be as high as 40 Gy, possibly due to the smaller genome and the lower body temperature compared to mammals [52]. For reactivation purposes, the 25 Gy dose was administered twice, which led to efficient reactivation of the mycobacterial disease, but also caused a 40% mortality *per se*, which is slightly less than the mortality caused by a single dose of 30 Gy [52]. Secondly, γ-radiation is likely to have direct effects on *M. marinum*. An aspect to be considered in the context of the reactivation model is the possibility of causing mutations in the bacterial genome, in addition to affecting the immune cell numbers of the host. *In vitro* studies have previously shown that *M. tuberculosis* is twice as radioresistant as *E. coli* [26]. Still, with doses above 1 Gy the viability of *M. tuberculosis* is adversely affected in a dose-dependent manner [26]. This has probably been the basis for the historical X-ray treatment *against* TB. Of note, also mutations advantageous to the bacteria can occur, and can be enriched in the population if a selection pressure, such as antibiotics, is applied. In our experiments, however, there was no selection pressure, but rather the pressure from the host's side was transiently relieved. Based on our FCM data, the dampened immune suppression of the host, rather than an advantageous mutation in mycobacteria, is likely to be the trigger for reactivation. Still, as a precaution, special measures should be taken to prevent the release of irradiated mycobacteria into the environment.

In conclusion, we have set up a system in which a latent mycobacterial disease can be established and assessed in adult zebrafish. The majority of the bacteria present in zebrafish enter a dormant state, with a smaller bacterial population remaining active. We were also able to induce a transition from this stable state to a progressive mode using repeated γ-irradiation mimicking immune suppressive states that cause human TB to reactivate. The reactivated infection is characterized by the absence of dormant mycobacterial population, similarly to the active primary disease preceding latency. Currently, there is no vaccine that would give proper protection against the reactivation of the latent disease. Gamma radiation induced reactivation should be an applicable model for testing new vaccine candidates, as the T cells required for protective immunity against TB are resistant to γ-radiation [53]. Thus, this proof-of-concept model for the reactivation of latent TB in a non-mammalian vertebrate shows high promise as a tool for large-scale studies on the related mechanisms.

## Materials and Methods

### Zebrafish lines and maintenance

For most experiments, adult (5–8 month-old) wild-type AB zebrafish were used. In addition, adult, rag1 (−/−) hu1999 mutant fish (from ZIRC) were used. For the FACS analysis, transgenic lines Tg(lck:lck-EGFP)∧cz2 and Tg (rag2:GFP)∧zdf8 (from ZIRC) were also used. Fish were kept in a flow-through system with a light/dark cycle of 14 h/10 h and were fed with SDS 400 food twice daily.

### Ethics statement

All experiments have been accepted by the Animal Experiment Board in Finland (under the Regional State Administrative Agency for Southern Finland) and were carried out in accordance with the EU-directive 2010/63/EU on the protection of animals used for scientific purposes and with the Finnish Act on Animal Experimentation (62/2006).

Licence for the zebrafish facility: LSLH-2007-7254/Ym-23, Licence for experiments: ESLH-2008-07610/Ym-23 and 20.10.2010 ESAVI-2010-08379/Ym-23.

### Experimental infection

*M. marinum* (ATCC 927) was cultured similarly as described in [10] with the following modifications: culture at 29°C, concentration of Tween 80 0.2%. Bacteria were first cultured on plates for 1 wk, transferred into liquid medium for 4 d, diluted once ∼1∶10, cultured to an OD600 of 0.495–0.680, collected by centrifugation and diluted appropriately with sterile 0.2 M KCl +0.3 mg/ml phenol red (Sigma-Aldrich). For qPCR experiments PBS without phenol red was used. The fish were briefly anesthetized in 0.02% 3-aminobenzoic acid ethyl ester (pH 7.0) (Sigma-Aldrich) and intraperitoneally (i.p.) injected with 5 µl using an Omnican 100 30 G insulin needle (Braun, Melsungen, Germany). To verify the bacterial dose, samples of bacterial dilutions were taken while infecting, diluted when needed and plated onto 7H10 plates. The low dose was 34±15 cfu and the high dose 2029±709 cfu. In survival experiments, humane end point criteria approved by a national ethical board were followed. If any of the following criteria were fulfilled, the animals were euthanized: lack of response to touch, abnormal swimming, gasping, observable swelling, observable waisting or loss of scales.

### Histology

Fish were euthanized by incubation in 0.04% 3-aminobenzoic acid ethyl ester (Sigma-Aldrich) pH 7.0. Heads and tails were removed and the fish were fixed in 10% phosphate buffered formalin pH 7.0 for 5–11 days at RT. After 1 week of de-calcification with 20% EDTA-citrate pH 7.2 samples were rinsed with tap water, transferred through an ethanol series with increasing concentrations, put into xylene and longitudinally embedded in paraffin. 5 µm sections were cut; every 40^th^ section was placed on a slide. The fish were sectioned thoroughly so that the entire kidney tissue lining the spine was included. The slides were stained with Ziehl-Neelsen staining and analyzed using the 200× magnification of an Olympus BX51 microscope. For Mallory's trichrome staining standard methods were used.

### qPCR

DNA extraction from mycobacteria: For determination of the bacterial load, the peritoneal cavity of the euthanized fish was emptied. The organs were put into weighed metal bead containing homogenization tubes (Mobio, California, USA) and frozen at −80°C. The mass of the organ sample was determined. The self-prepared modified enzymatic lysis buffer MELB (20 mM Tris-HCl pH 8.0, 20 mM sodium EDTA pH 8.0, 1.2% Triton X 100) was added to the samples, which were homogenized in a volume of 575 µl using the PowerLyzer24 (Mobio) at speed 3,200 for 3×20 second cycles with 30 second pauses. An appropriate proportion (sample mass <25 mg) of the homogenate was taken for DNA extraction. The samples were sonicated in an m08 water bath sonicator (Finnsonic, Lahti, Finland) for 9 min. Lysozyme (Sigma-Aldrich) was added to a final concentration of 20 mg/ml and incubated at 37°C for 2 h. After incubation, MELB was added to the samples to equalize the volume of all samples to 180 µl. From this point on the QIAGEN DNeasy Blood & Tissue Kit manufactures protocol for DNA extraction from gram-positive bacteria was used. The DNA was eluted twice with a volume of 200 µl.

RNA-DNA co-extraction from infected zebrafish: Organs were collected as above, and homogenized in tubes with ceramic beads, 3200 rpmi, 3×40 s in 1.5 ml of TRI reagent (MRC, OH, USA). RNA extraction was carried out according to the manufacturer's protocol. DNA was extracted from the same sample for determination of the mycobacterial load by adding back extraction buffer (1∶1) (4 M guanidine thiocyanate (Sigma-Aldrich), 50 mM sodium citrate, 1 M Tris) on top of the lower phase after phenol-chloroform phase separation. DNA was thereafter precipitated with isopropanol, washed with ethanol twice and dissolved in sterile ddH_2_O.

Primers were designed for the *M. marinum* 16S–23S ITS sequence: F: 5′-caccacgagaaacactccaa-3′ R: 5′-acatcccgaaaccaacagag-3′. For quantification of mycobacterial load SENSIFAST NO-ROX SYBR was used. The final reaction solution had the following composition: 1× SENSIFAST NO-ROX SYBR GreenPCR Master Mix (stock 2×), 0.4 µM MMITS1 forward primer, 0.4 µM MMITS1 reverse primer 3 µl of template. Duplicate or triplicate dilutions were made for each sample. A standard curve was made by extracting the total DNA from a known amount of bacteria (logarithmic culture) and 10-fold using serial dilutions. DNA extracted from three healthy fish were included in order to determine the background signal. The qPCR was carried out using the BIO-RAD CFX96 cycler with the following settings: 1. 3 min 95°C, 2. 5 s 95°C, 3. 10 s 65°C, 4. 5 s 72°C, 5. 39 cycles from 2. to 4. 6. Melting curve 55–95°C at 0.5 intervals.

For q-RT-PCR, primer sequences can be found in the Supporting Information (Text S2). For gene-expression measurements, Bio-Rad iScript One-Step RT-PCR Kit with SYBR Green was used according to the manufacturer's instructions. The optimal annealing temperature of each primer pair were determined using melting curve analysis and agarose gel electrophoresis. The expression of *glyceraldehyde 3-phosphate dehydrogenase* (*GAPDH*, ZDB-GENE-030115-1) was used for normalization of the host genes. The results from mycobacterial dormancy genes were normalized to the bacterial load measured from the corresponding DNA sample.

Bio-Rad CFX Manager software and GraphPad Prism 5.02 were used in the analysis. Using the standard curve, a concentration in units of bacterial genome copies was obtained for every sample. The bacterial load per fish (the visceral organs) could be calculated: (qPCR result (bact./µl of template) x qPCR sample dilution factor Y x total DNA eluate volume (µl) x homogenate dilution factor X). The limit of detection with the qPCR method was estimated to be ∼10^3^ cfu/fish.

### Plating on antibiotic plates +/− Rpf

For production of secreted Rpf for resuscitation of dormant mycobacteria, *Micrococcus luteus* was cultured. An inoculate from a glycerol stock of *M. luteus* was revived in 10 ml of LB liquid medium at 37°C o/n in rotation (to an OD605 = 0.100). A 100 ml volume of lactate minimal medium, LMM (composition described in [54] with the exception of a lower concentration of lactate (0.5% w/v) [24] was inoculated with 4 ml of the o/n culture and was cultured aerobically in rotation (150 rpm) at 30°C for 4 days to an OD605 = 0.705. After centrifugation (10,000 g, 3 min) the supernatant was sterile filtered and aliquots were stored at −80°C. For Rpf plating an aliquot was thawed and 500 µl was absorbed on each 7H9 antibiotic plates. On –Rpf plates, 500 µl of fresh, sterile LMM was absorbed. The concentrations of antibiotics on plates were as described in [55] and 20 µg/ml azithromycin (Sigma-Aldrich). Fish were homogenized with the same settings as for DNA extractions from mycobacteria in sterile PBS supplemented with 0.5% Tween 80 (v/v). Dilutions were plated +/− Rpf and incubated in the dark at 25°C for 15–17 days. *M. marinum* colonies were counted and the average load of culturable bacteria was determined. As controls, active logarithmic *M. marinum* broth cultures and old stationary *M. marinum* broth cultures that had been kept in closed bottles at +29°C for 5–8 months were plated +/− Rpf and cultured as described above.

### Immunosuppression

Irradiation with 25 Gy was carried out with Gammacell 1000 irradiator in glass flasks with 5 fish/80 ml of water. In the reactivation experiments, low-dose infected fish were irradiated twice with one month between the doses. Non-infected controls were similarly irradiated.

### Flow cytometry (FCM)

Nine days after irradiation, fish were euthanized and the kidneys were collected and placed into PBS supplemented with 1% fetal calf serum (FCS) on ice. In the first experiment (1 wk post irradiation) kidneys from each group of fish (4/group) were pooled. Fish were analyzed individually in the second one (5 weeks post 1.irradiation). Also kidneys from untreated groups of the same fish lines were similarly collected in order to determine a baseline. Kidneys were homogenized by pipetting the entire volume (1 ml) up and down 15 times. The samples were also filtered before analysis. Immune cell populations were determined using a FACSCantoII (Beckton Dickinson) and the FACSDiva software. The results were analyzed using FlowJo (Treestar Inc, Ashland, OR). Lymphocytes, blood cell precursors, erythrocytes and granulo/monocytes were identified based on the cellular granularity (SSC-A) and size (FSC), (see Figure S2) according to [56]. In addition, reporter fish lines Lck-GFP (T lymphocytes) and RAG-GFP (B and T lymphocytes) we used in some experiments to further confirm the identity of the immune cell populations. Total 30,000 events per sample were collected for analysis.

### Statistical analysis

Statistical analysis was carried out using the GraphPad Prism software (5.02). For determination of statistical significance of differences in bacterial loads, number of granulomas, affected organs and leukocyte counts and gene-expression data, a non-parametric one-tailed Mann-Whitney test was used. In survival experiments, the log-rank Mantel-Cox test was used. In Rpf experiments, the plates with the same sample +/− Rpf were compared pair-wise, and a one-tailed paired t-test was used. P-values<0.05 were considered significant.

### List of genes mentioned in the article

**Zebrafish (** ***Danio rerio*** **):**

*Nos2b*: ZDB-GENE-080916-1

*IFNγ1-2*: ZDB-GENE-040629-1

*TNFα*: ZDB-GENE-050317-1

*IL-1β*: ZDB-GENE-040702-2

*IL-6*: ZDB-GENE-120509-1

*IL-12*: ZDB-GENE-060724-1

*GAPDH:* ZDB-GENE-030115-1

**M. marinum:**

*GltA1*: MMAR_1381

*HspX*: MMAR_3484

*DevR*: MMAR_1516

*GltA1*: MMAR_1381

*Tgs1*: MMAR_1519

## Supporting Information

Figure S1 **qPCR and plating give similar results.** Dilutions (1, 1∶10, 1∶1000) of mycobacterial culture (logarithmic growth phase) were added onto healthy fish organ samples. The amount of bacteria added was determined by plating dilutions of the culture (result shown as white bars). The samples were homogenized and the DNA was extracted. The bacterial concentration was determined by qPCR (result shown as black bars).
(TIF)

Figure S2 **A single 25 Gy dose of gamma radiation is not sufficient for reactivation of latent tuberculosis.** Latently infected adult zebrafish (n = 39) were γ-irradiated (25 Gy). Latently infected, non-irradiated zebrafish (n = 25) were used as controls. The effects of the irradiation were controlled by irradiating non-infected fish (n = 30). (A) Survival was followed for 28 days. * P<0.05 (B) To determine the bacterial load, 5 fish were collected 2 weeks after irradiation. Similarly infected non-irradiated controls were also collected.
(TIF)

Figure S3 **Gamma irradiation depletes the lymphocyte population in adult zebrafish.** 4 groups (1 Tg(rag2-GFP). 1 Tg(lck:lck-egfp) and 2 wt groups) of 4 adult zebrafish were γ-irradiated with 25 Gy or left untreated. Kidneys were collected 9 d post irradiation, pooled and analyzed by FCM. FSC-SSC –plots were gated based on [56] as follows: E = erythrocytes, G/M = granulocytes & monocytes, L = lymphocytes, P = blood cell precursors. The numbers by the gates show the percentage of cells within the gate of the total live population. For GFP-expressing lines (rag2 and lck) a GFP gate was also used. The GFP positive populations were reanalyzed on a FSC-SSC -plot. The lymphocyte population was most severely affected by irradiation, whereas the number of granulo/monocytes decreased less. An increase in the proportion of blood cell precursors was detected. A reanalysis of the GFP results verified that the GFP-expressing cells were mostly present within the lymphocyte gate.
(TIF)

Text S1 **A qPCR-assay for quantifying of** ***M. marinum*** **load in adult zebrafish tissues.**
(DOC)

Text S2 **Sequences of the Q-RT-PCR primers used in the study.**
(DOC)
