# Supplementary figures and images for: Mycobacterium marinum Causes a Latent Infection that Can Be Reactivated by Gamma Irradiation in Adult Zebrafish
Source: PLoS Pathog. 2012 Sep 27;8(9):e1002944. doi: 10.1371/journal.ppat.1002944 (PMC3459992; doi:10.1371/journal.ppat.1002944)

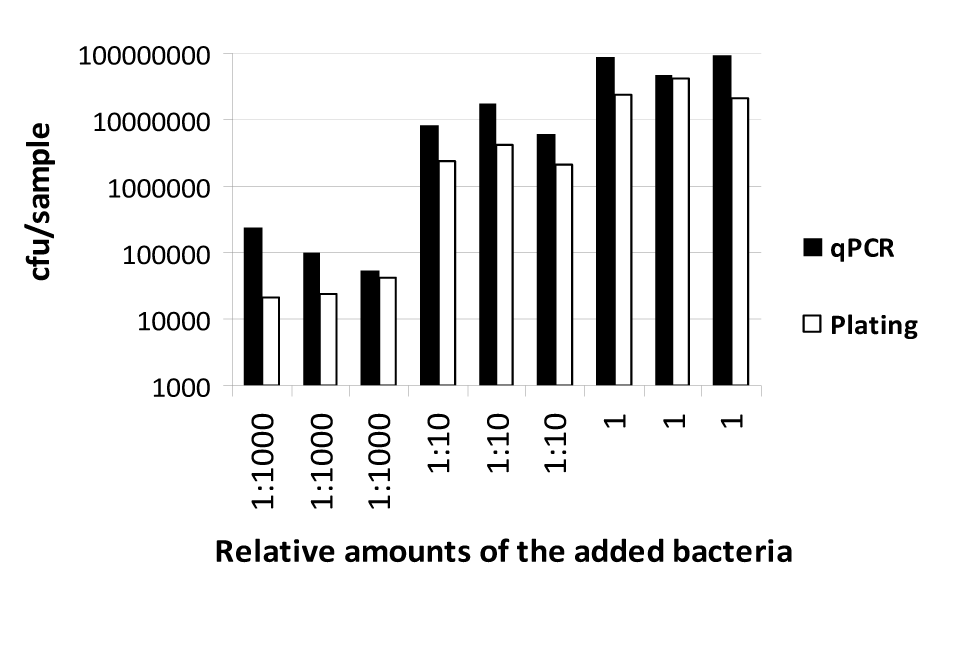

Supplement: Figure S1 — qPCR and plating give similar results. Dilutions (1, 1∶10, 1∶1000) of mycobacterial culture (logarithmic growth phase) were added onto healthy fish organ samples. The amount of bacteria added was determined by plating dilutions of the culture (result shown as white bars). The samples were homogenized and the DNA was extracted. The bacterial concentration was determined by qPCR (result shown as black bars). (TIF) [file ppat.1002944.s001.tif]

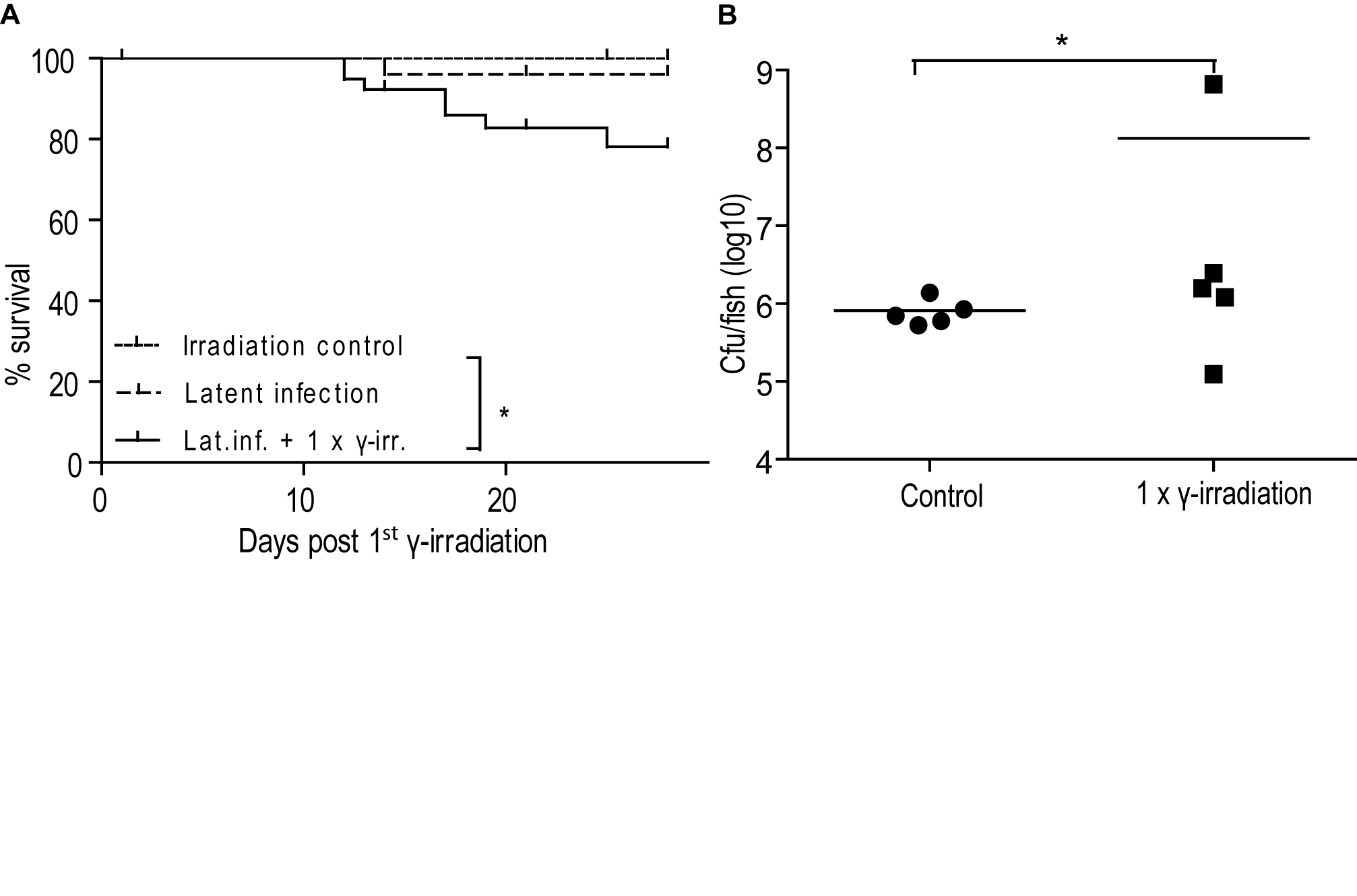

Supplement: Figure S2 — A single 25 Gy dose of gamma radiation is not sufficient for reactivation of latent tuberculosis. Latently infected adult zebrafish (n = 39) were γ-irradiated (25 Gy). Latently infected, non-irradiated zebrafish (n = 25) were used as controls. The effects of the irradiation were controlled by irradiating non-infected fish (n = 30). (A) Survival was followed for 28 days. * P<0.05 (B) To determine the bacterial load, 5 fish were collected 2 weeks after irradiation. Similarly infected non-irradiated controls were also collected. (TIF) [file ppat.1002944.s002.tif]

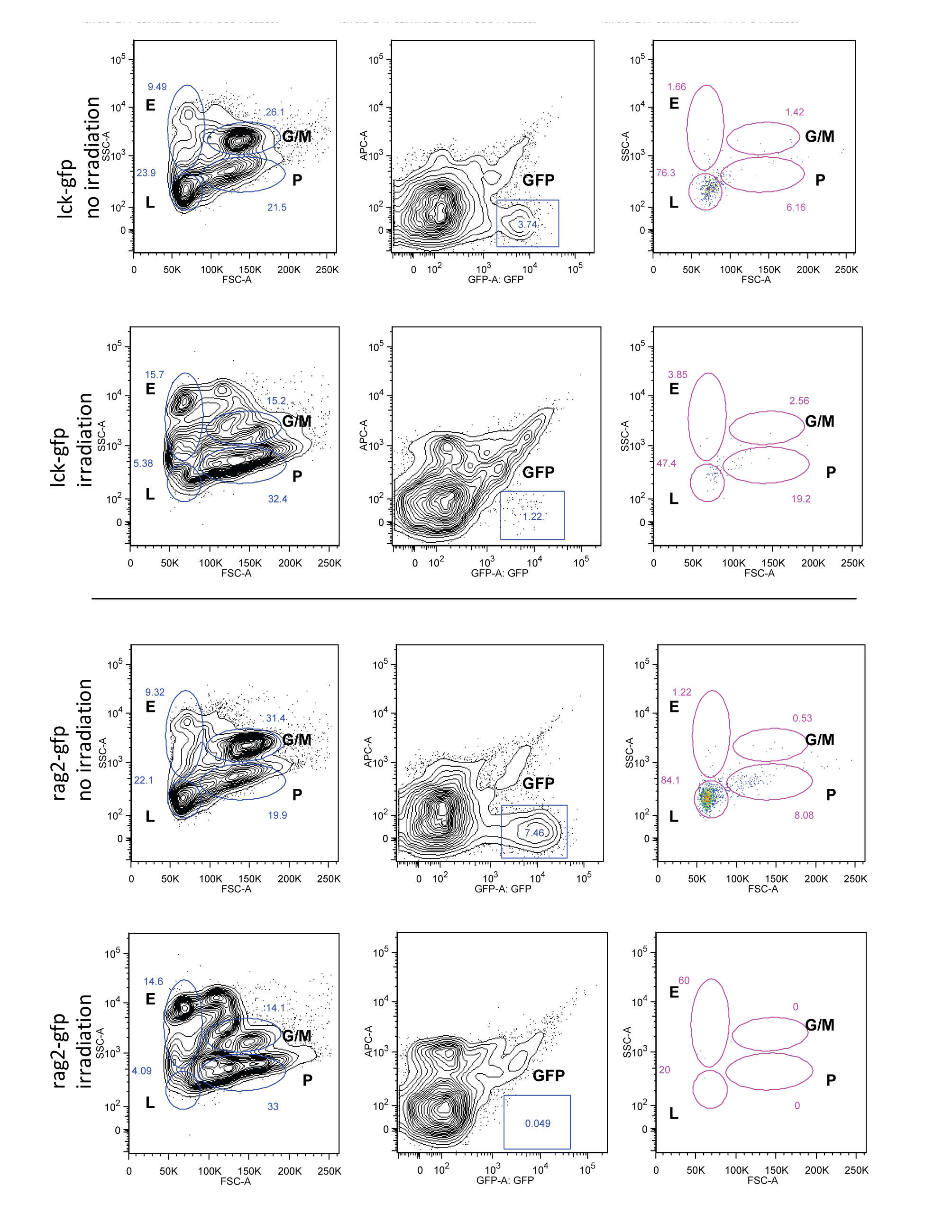

Supplement: Figure S3 — Gamma irradiation depletes the lymphocyte population in adult zebrafish. 4 groups (1 Tg(rag2-GFP). 1 Tg(lck:lck-egfp) and 2 wt groups) of 4 adult zebrafish were γ-irradiated with 25 Gy or left untreated. Kidneys were collected 9 d post irradiation, pooled and analyzed by FCM. FSC-SSC –plots were gated based on [56] as follows: E = erythrocytes, G/M = granulocytes & monocytes, L = lymphocytes, P = blood cell precursors. The numbers by the gates show the percentage of cells within the gate of the total live population. For GFP-expressing lines (rag2 and lck) a GFP gate was also used. The GFP positive populations were reanalyzed on a FSC-SSC -plot. The lymphocyte population was most severely affected by irradiation, whereas the number of granulo/monocytes decreased less. An increase in the proportion of blood cell precursors was detected. A reanalysis of the GFP results verified that the GFP-expressing cells were mostly present within the lymphocyte gate. (TIF) [file ppat.1002944.s003.tif]
