# Supplementary material for: Mycobacterium marinum Causes a Latent Infection that Can Be Reactivated by Gamma Irradiation in Adult Zebrafish
Source: PLoS Pathog. 2012 Sep 27;8(9):e1002944. doi: 10.1371/journal.ppat.1002944 (PMC3459992; doi:10.1371/journal.ppat.1002944)
Supplement: Text S1 — A qPCR-assay for quantifying of M. marinum load in adult zebrafish tissues. (DOC) [file ppat.1002944.s004.doc]

**Text S1. A qPCR-assay for quantifying of *M. marinum* load in adult zebrafish tissues**

Plating is a routinely used method for bacterial quantification in biological samples. *M. marinum* is naturally resistant to a wide range of antibiotics (55) and plating on multiantibiotic 7H10 plates is a published method for quantifying of *M. marinum* from zebrafish samples (9, 56). However, in our experiments, the bacterial load could not be determined from all individuals, as part of the zebrafish population harbored fast-growing commensal bacteria that grew on the plates despite antibiotics. In order to consistently be able to determine the load in every individual, an *M. marinum* specific qPCR method was developed. Also, the qPCR method allowed the quantification of the total mycobacterial load in the fish, irrespective of the replicative status of the bacterium. The qPCR could be compared with the plating results to determine the proportion of dormant bacteria.

The *M. marinum* 16S–23S ITS (internal transcribed spacer) region in the rRNA operon was chosen as the target for amplification as it represents a highly species specific sequence allowing reliable identification (Gurtler & Stanisich, 1996, Rot*h et a*l. 1998). This target was also a convenient choice as it is present as one copy per *M. marinum* genome ( Rot*h et a*l. 1998) and is thus a direct indicator of the amount of the bacteria in the sample material. Possible targets in other species were analyzed using the Primer Blast. Unsurprisingly, a *Mycobacterium ulcerans* sequence was also recognized by the primers. *M. ulcerans* is the closest relative to *M. marinum* and the two species cannot be distinguished from one-another even by 16S-23S ITS sequence (Rot*h et a*l. 1998). *M. ulcerans* has not been reported to infect fish and is not expected to be present in the healthy fish population of our research facility. Thus it was not thought to pose a problem in these experiments.

In our experiments, a single product of 142 bp was amplified in the infected fish samples. To validate the method, the experiment described in Figure S1 was carried out. The bacterial load determined by qPCR from samples extracted from fish tissues and added bacteria seemed consistently higher (on average 4-fold) than determined by plating. This is the general error of the method. However, the order of magnitude of the load was the same for both methods, and there was a correlation between the loads determined by plating and the qPCR method. The qPCR method allowed consistent and reliable quantification of the mycobacterial load in the zebrafish viscera, and was found to be superior to quantitative plating, in which contaminating drug-resistant bacterial species in the fish flora frequently cause problems.

**References**

Gurtler, V & Stanisich, V.A. 1996, Microbiology, vol. 142, no. 1, pp. 3-16.

Roth, A. *et al.* 1998, Journal of clinical microbiology, vol.36, no. 1, pp. 139-147.
