# Supplementary material for: Mycobacterium marinum Causes a Latent Infection that Can Be Reactivated by Gamma Irradiation in Adult Zebrafish
Source: PLoS Pathog. 2012 Sep 27;8(9):e1002944. doi: 10.1371/journal.ppat.1002944 (PMC3459992; doi:10.1371/journal.ppat.1002944)
Supplement: Text S2 — Sequences of the Q-RT-PCR primers used in the study. (DOC) [file ppat.1002944.s005.doc]

**Q-RT-PCR primers**

*Danio rerio*

**IFNg1-2**

F: GGGCGATCAAGGAAAACGACCC

R: TAGCCTGCCGTCTCTTGCGT

**IL-12**

F:AGCATGGCTCTGGCTCTGGC

R: TGCTCCTTCATCTTTCCCTCCTTCT

**Nos2b**

F: TCACCACAAAAGAGCTGGAATTCGG

R: ACGCGCATCAAACAACTGCAAA

**GAPDH**

F: AGTGTCAGGACGAACAGAGGCT

R: GCCAATGCGACCGAATCCGTTA

*M.marinum*

**GltA1**

F: CCACAGCCACATGAGTTACG

R: GCTCGAAGGTATCCACAACC
